# S98. THE RELATIONSHIP BETWEEN CARDIOVASCULAR RISK FACTORS AND COGNITIVE IMPAIRMENT IN PEOPLE WITH SCHIZOPHRENIA: A SYSTEMATIC REVIEW AND META-ANALYSIS

**DOI:** 10.1093/schbul/sby018.885

**Published:** 2018-04-01

**Authors:** Katsuhiko Hagi, Tadashi Nosaka, Christoph Correll

**Affiliations:** 1 Dainippon Sumitomo Pharma; 2 Hofstra Northwell School of Medicine

## Abstract

**Background:**

Schizophrenia is associated with cardiovascular abnormalities, including diabetes mellitus (DM), metabolic syndrome (MetS), obesity, hypertension and dyslipidemia. Since cardiovascular risk factors can worsen cognition in the general population, they may also contribute to cognitive impairment in schizophrenia.

**Methods:**

Performing an electronic search in Embase/Scopus/MEDLINE/PubMed/Cochrane library/www.clinicaltrials.gov), we meta-analyzed cross-sectional studies comparing neurocognitive functioning in schizophrenia patients with versus without cardiovascular risk factors. Global cognition and Attention/Vigilance, Reasoning/Problem Solving Speed of Processing Verbal Learning, Visual Learning and Working Memory were analyzed.

**Results:**

Data from 22 trials (n=9,579, DM=8 studies, MetS=9 studies, obesity=8 studies, overweight=8 studies, arterial hypertension=5 studies, dyslipidemia=4 studies) were meta-analyzed.

Significantly greater global cognitive deficits in schizophrenia were associated with presence of DM (n=2,976, Hedges’ g=0.322; 95%CI=0.227–0.417, p<0.001), MetS (n=2,269, Hedges’ g=0.409; 95%CI=0.166–0.652, p=0.001) and hypertension (n=1,899, Hedges’ g=0.210; 95%CI=0.110–0.311, p<0.001), but not with obesity (n=2,779, Hedges’ g=0.695, 95%CI=-0.320, 1.709, p=0.180), overweight (n=2,825, Hedges’ g=0.406; 95%CI=-0.445, 1.257, p=0.350), or dyslipidemia (n=1,761, Hedges’ g=-0.055; 95%CI=-0.162, 0.051, p=309). Among 6 analyzed cognitive domains, DM (Hedges’ g=0.23–0.40) and hypertension (Hedges’ g=0.15–0.27) were each associated with significantly greater cognitive dysfunction in 4 domains, MetS with 3 (Hedges’ g=0.16–0.42), obesity (Hedges’ g=0.35) and overweight (Hedges’ g=0.24) with one, and dyslipidemia with none.

**Discussion:**

DM, MetS and hypertension are associated with significant global cognitive impairment in schizophrenia. The same cardiovascular risk factors and, less so, obesity and overweight, are associated with worse performance in specific cognitive domains. Research is needed to determine to what degree improving cardiovascular risk factors also improves cognitive impairment in schizophrenia.

